# Non-linear Characterization of Commercial and Decellularized Hydrogels: Statistical Framework Enhanced by Bayesian Optimization

**DOI:** 10.1007/s12195-026-00913-1

**Published:** 2026-06-30

**Authors:** D. E. García-García, D. Marques, H. Amaveda, M. Mora, J. Asín, I. Villaoslada, P. M. Baptista, M. A. Pérez, J. M. García-Aznar

**Affiliations:** 1 Multiscale in Mechanical and Biological Engineering, Instituto de Investigación en Ingeniería de Aragón (I3A), University of Zaragoza, 50014 Zaragoza, Spain; 2 Instituto de Nanociencia y Materiales de Aragón (INMA), CSIC-Universidad de Zaragoza, 50018 Zaragoza, Spain; 3 Department of Statistical Methods, University Institute of Mathematics (IUMA), University of Zaragoza, 50009 Zaragoza, Spain; 4 Instituto de Investigación Sanitaria de Aragón (IIS Aragón), 50009 Zaragoza, Spain; 5 Biomedical Engineering Department, Carlos III University of Madrid, Madrid, Spain; 6 Biomedical Research Networking Center in Hepatic and Digestive Diseases (CIBERehd), Madrid, Spain; 7 ARAID Foundation, Zaragoza, Spain

**Keywords:** Computational mechanical characterization, Bayesian optimization, Statistical analysis, Hyperelasticity, Inter- and intra-sample variability

## Abstract

****Scope**:**

Hydrogels are widely used in the design of tissue substitutes because of their ability to mimic the extracellular matrix (ECM). Their mechanical cues critically influence the cellular response, making accurate characterization essential. However, it remains challenging due to their intricate nature. This study computationally evaluates the hyperelastic properties of next-generation hydrogels of high biomedical interest, including basal membrane extract and decellularized liver matrices, as well as structural proteins.

****Methods**:**

We present a combined framework based on Bayesian optimization and statistical analyses that go beyond classical least-squares fitting, leveraging rheological experimental data. It defines each hyperelastic strain-energy density function, and addresses both intra- and inter-sample variability. This approach quantifies uncertainty and reveals the natural variability that deterministic models overlook, and it also enables quantification of coefficient variation with composition.

****Results**:**

Validation against experimental data shows computational fits of 5% error in most cases, and low calculation time. Analyses reveal that composition—collagen addition, fibrin concentration, and decellularized extracellular matrix (dECM) age—modulate initial stiffness, nonlinearity, and overall mechanical resistance of the hydrogels.

****Conclusion**:**

Hydrogels derived from basal membrane extracts exhibit comparable non-linear mechanics, while collagen addition reduces stiffness and nonlinearity. In fibrin-based hydrogels with decellularized liver matrix, mechanical behavior is concentration and age-dependent. Such insights are highly relevant for mechanobiology, enabling prediction of how cells sense mechanical cues and scaffold composition influences in vivo interactions.

**Supplementary Information:**

The online version of this article (10.1007/s12195-026-00913-1) contains supplementary material, which is available to authorized users.

## Introduction

Tissue engineering (TE) has emerged as a response to the shortage of organs needed for the treatment of trauma and tissue degeneration [1]. In this field, biomaterial substrates are essential to mimic the ECM, highlighting hydrogels as three-dimensional (3D) networks of cross-linked polymers capable of absorbing large amounts of water or biological fluids [2]. Since their first application as contact lenses in the 1960s, their versatility has enabled their use in the recreation of organs such as liver, kidney, and lung [3]. Other studies have reported their application as wound [4] and tumor models, like Alamán-Díez et al. [5], that provide a framework to analyze the impact of mechanical properties on cancer growth and signaling pathways.

Hydrogels combine high biocompatibility, controllable biodegradability, and efficient molecular transport within their structure [6]. Their injectability reduces the invasiveness of medical procedures and enables adaptation to irregular defects such as cartilage lesions [7]. Additionally, their 3D architecture provides a more physiologically relevant environment than two-dimensional (2D) cultures, which can alter cell behavior [8, 9]. This 3D context has been shown to promote embryonic stem cell differentiation [10, 11], for example, Kaur et al. [12] demonstrated that intrahepatic cholangiocyte organoids (ICOs) encapsulated in dECM liver hydrogels engrafted and differentiated into hepatocytes, while reducing fibrosis.

From a mechanical perspective, the polymeric network architecture and the individual filaments’ characteristics determine the properties of hydrogels, making them highly controllable and adaptable [13, 14]. They are characterized by an aqueous microenvironment and a nonlinear viscoelastic behavior [15]. Their biological relevance is highlighted by the fact that cellular processes such as differentiation, fibrosis, and malignancy are regulated by substrate mechanics. However, replicating the mechanical properties of biological tissues remains a significant challenge, largely due to their inherent variability and complexity resulting from bidirectional interactions with cells [16]. These interactions are highly dynamic, time-dependent, and influenced by multiple factors, including ECM composition and biomechanical signaling [17]. In fact, studies support that bioresponsive scaffolds are more easily manipulated by cells [18].

Despite advances, computational models still face limitations in accurately capturing these properties. To model the complex behavior of hydrogels, mathematical approaches are broadly classified into single-phase and multiphase models [19]. Single-phase models treat hydrogels as a continuum where multiple components coexist, whereas multiphase models represent hydrogels as systems composed of distinct phases with specific properties, increasing numerical complexity [20, 21]. Among multiphase approaches, polymeric network models have been developed, as this structure predominantly governs the mechanical response of hydrogels [22]. These approaches aim to bridge microstructural organization with overall mechanical properties [23], employing frameworks such as the *worm-like chain* (WLC) model [24] and its adaptations. Valero et al. [25] extended this model to analyze the effects of fiber concentration and crosslinker addition. Between single-phase approaches, poroviscoelastic theory has gained particular attention [26]. Additionally, finite element (FE)-based methods incorporating nonlinear constitutive equations have been developed and validated through experimental techniques such as atomic force microscopy [27]. Due to the polymeric nature of hydrogels, hyperviscoelastic models are particularly suitable [28]. These models combine hyperelastic energy density functions, $\psi$, with Prony series and are experimentally validated via uniaxial/biaxial tests for the elastic component, and stress relaxation/creep tests for the viscoelastic behavior, as demonstrated by Pérez-Benito et al. [29]. Some approaches also extend the analysis, for instance by accounting for the water content [30], or to capture the self-healing behavior [31]. In certain cases, the combination of $\psi$ with Prony series has been employed to enhance accuracy [32].

We present a physically and statistically grounded modeling framework to capture the mechanical response of hydrogels while accounting for intra- and inter-material variability. This also enables a quantitative link between composition and mechanical behavior, an aspect still poorly understood, as highlighted by Mawazi et al. [33]. The primary objective of this study is to computationally characterize the hyperelastic behavior of next-generation hydrogels increasingly used as scaffolds in TE. To this end, we fit hyperelastic constitutive models to experimental stress–strain data using a Bayesian [34] optimization algorithm based on an iterative scheme. This framework is further supported by statistical analyses to account for intra-material variability inherent to biological materials and inter-material variability to identify the mechanical response modulated by composition. Our experimental data suggest that the viscous contribution is limited under the loading conditions considered, leading to a predominantly elastic mechanical response.

The remainder of this paper is structured as follows. “Materials and Methods” section delineates the methodology and is divided into two parts. An experimental part, covering the synthesis of the hydrogel formulations and their rheological characterization, and a computational framework that includes data preprocessing, Bayesian optimization algorithm for constitutive coefficient quantification, and statistical analysis. “Results” section presents the results, including the outcomes of the optimization process, the assessment of intra-material variability, and the statistical evaluation of how composition modulates mechanical properties. Finally, “Discussion” and “Conclusion” sections provide the discussion and conclusions, highlighting the implications of the findings in the context of mechanobiology and TE, as well as the proposed workflow.

## Materials and Methods

**Fig. 1:**
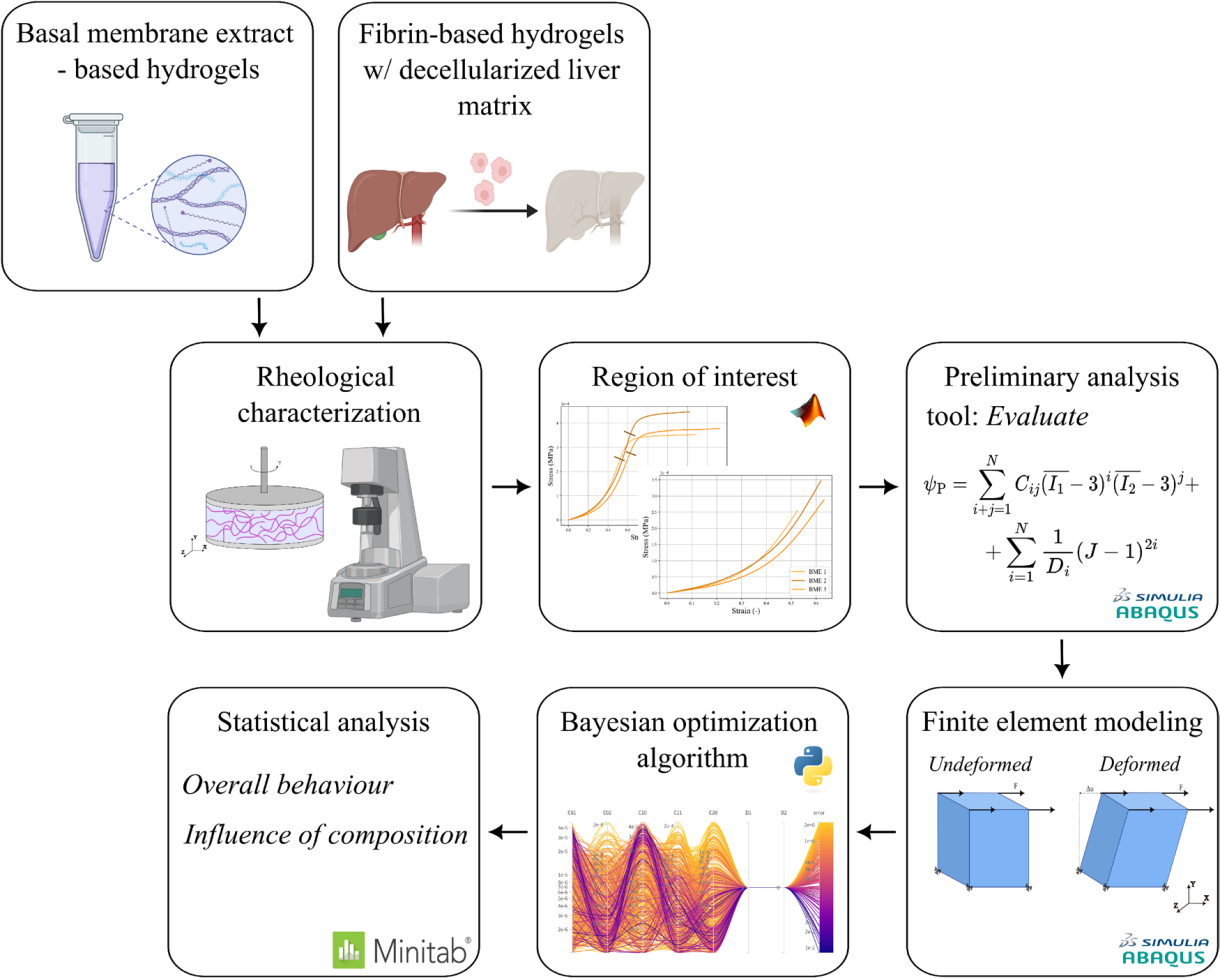
Workflow diagram illustrating the main steps performed. Sample preparation includes basal membrane extract-based hydrogel formulations (BME, Matrigel and BME combined with collagen type I), as well as fibrin-based hydrogels at 3 mg/mL and 5.75 mg/mL concentrations. The last ones are also combined with decellularized liver matrices, fetal or adult, obtained through a decellularization process. Each material is characterized by rheology tests. Rheological data are preprocessed to isolate the region of interest, corresponding to elastic behavior. A preliminary evaluation identifies the most suitable $\psi$ to describe the mechanical behavior of the hydrogels, the second-order (N = 2) Polynomial function. Next, the simplified FEM proposed by Valero et al. [25] is implemented to compute the stress–strain curves, applying a displacement representing half of the loading cycle. To identify the coefficients of the N = 2 Polynomial function that describes each experimental behavior, a Gaussian-process-based Bayesian optimization is employed. Finally, two statistical analyses to address intra- and inter-material variability of the hydrogels are conducted

Biologically derived hydrogels are synthesized, fabricated, and prepared for mechanical rheology testing, from which stress–strain curves are obtained to characterize their mechanical behavior. These data serve as the basis for computational analyses, including data preprocessing, FE modeling, coefficient optimization through a customized Bayesian optimization workflow, and statistical evaluation. The overall workflow of the study is illustrated in Fig. 1, whose steps will be developed in detail throughout this section.

### Synthesis and Fabrication of Hydrogels

This section describes how the different hydrogel formulations are prepared. We group them into two families based on their main components: basal membrane extract-based hydrogels and fibrin-based hydrogels with decellularized liver matrix. This classification is maintained subsequently.

#### Preparation of Basal Membrane Extract-Based Hydrogels

Basal membrane extract-based hydrogels include three formulations that share the same core components. The formulations are basement membrane extract (BME), Matrigel and BME enriched with collagen type I.

For their respective experiments, both BME Type (Cultrex) and Matrigel (Corning) are aliquoted and used undiluted. In addition, to prepare the BME with collagen type I mixture, rat tail collagen type I (Corning) is diluted in Advanced DMEM/F-12 (Gibco) with 50% BME of the total volume and $10\times$ Dulbecco’s phosphate-buffered saline (DPBS, Sigma-Aldrich). The final mixture reaches a collagen concentration of 2.5 mg/mL. Once the solution is prepared, the pH is adjusted to 7.4 using 0.5 M NaOH (Sigma-Aldrich).

#### Preparation of Fibrin-Based Hydrogels with Decellularized Liver Matrix

Six fibrin-based hydrogel formulations are prepared, varying in fibrin concentration (3 or 5.75 mg/mL) and in the origin of the dECM derived from fetal (FDM) or adult (ADM) pig liver. The formulations comprise fibrin hydrogels at both concentrations and fibrin-based hydrogels with dECM by combining each fibrin concentration with either fetal or adult matrix. Preparation of these hydrogels requires a multistep experimental process described below.

*(I) Isolation and decellularization of pig livers*

The decellularization process for adult livers is as follows. First, livers from adult pigs (from the local slaughter house) are surgically extracted, shortly perfused with phosphate-buffered saline (PBS) to remove excess blood, and subsequently frozen at $-30\,^{\circ}$C. After thawing, the livers are cannulated and perfused through the portal vein, hepatic artery, and vena cava with deionized water (dH$_{2}$O) for 2 h. Decellularization is performed by perfusing 25–30 L of a 1% (w/v) sodium dodecyl sulfate (SDS; Sigma, L5750) solution in dH$_{2}$O containing 0.1% (v/v) ammonium hydroxide. Finally, the tissue is rinsed by perfusing dH$_{2}$O at twice the volume of SDS.

As for fetal liver matrices, they are obtained from fetuses collected at gestational day 82 and frozen whole at $-30\,^{\circ}$C (IACUC protocol approval PI08/17). After thawing at $4\,^{\circ}$C, the liver is surgically removed, cannulated through the umbilical and portal veins, and perfused with dH$_{2}$O for 2 h. Decellularization is carried out by perfusing 2 L of a detergent solution composed of 1% (v/v) Triton X-100 (Panreac, A1388) and 0.1% (v/v) Ammonium Hydroxide (Panreac, 1411301612). Then it is washed by sequential perfusion with 500 mL of 3.4 M NaCl in dH$_{2}$O (Panreac, 131659), followed by 3–4 L of a 0.9% (w/v) NaCl in dH$_{2}$O solution.

In adult and fetal liver protocols, perfusion is performed at increasing flow rates to ensure effective decellularization.

*(II) Lyophilization of decellularized liver matrices*

Both adult and fetal decellularized livers are chopped into smaller fragments, frozen at $-80\,^{\circ}$C, and lyophilized using a Telstar LyoQuest freeze-dryer at 100 mbar. The resulting lyophilized matrices are then cryomilled into a fine powder by deep-freezing with liquid nitrogen, followed by mechanical work using a mortar and pestle. Alternatively, a cryomill is employed.

*(III) Enzymatic digestion of cryomilled decellularized liver matrix*

Cryomilled decellularized matrix are digested at 20 mg/mL in a dH$_{2}$O solution containing porcine pepsin (Sigma, 77151-1 G) at 1 mg/mL and 0.01 M HCl, under constant agitation at 1200 rpm for 48 h at $37\,^{\circ}$C. Digestion products are centrifuged at $4\,^{\circ}$ C at 10.000 $\times$ g for 10 min, and the supernatant is collected and frozen at $-30\,^{\circ}$C. The pellet is subjected to a second identical digestion, and its supernatant is also recovered and frozen. All digestion supernatants are lyophilized, sterilized with a 10 kGy dose of gamma radiation, and resolubilized in milliQ H$_{2}$O. Protein content is quantified using a bicinchoninic acid (BCA) assay (Thermo Scientific Pierce™ BCA Protein Assay Kit) according to the manufacturer’s protocol. Absorbance is measured with a Synergy HT (BioTek) Microplate Reader. Final dECM protein solutions are stored at $-80\,^{\circ}$C until use.

*(IV) Production of fibrin-based hydrogel with decellularized liver matrix*

Human plasma-derived fibrinogen (Merck, 341576-1GM) and thrombin (Merck, 605190-1000U) are dissolved in milliQ H$_{2}$O, aliquoted in small volumes, and stored at $-80\,^{\circ}$C. Fibrin-based hydrogels supplemented with decellularized liver matrix are prepared by mixing a master solution in milliQ H$_{2}$O containing MEM $1\times$ (ThermoFisher, 11430030), CaCl$_{2}$ at 40 mM, tranexamic acid (ThermoFisher, 228040500) at 160 µg/mL, decellularized liver matrix at 2 mg/mL of protein and hyaluronic acid at 2 mg/mL (Sigma, 53747-1G). The solution is neutralized using NaOH.

Subsequently, thrombin is added at a concentration of 2.2 U/mL, followed by the addition of fibrinogen at either 3 or 5.75 mg/mL, depending on the intended application. To prevent fibrinogen precipitation, it is kept at either $-80\,^{\circ}$C or $37\,^{\circ}$C before use. All other components are handled on ice to delay enzymatic activity during preparation.

### Rheological Measurements

The mechanical properties of the hydrogels are assessed with a stress-controlled rheometer (Haake Rheostress 1, Thermo Fisher Scientific, Waltham, MA, USA). An oscillatory shear stress ($\tau = \tau _{0} \sin (\omega t)$) is applied to the sample, recording the resulting oscillatory strain ($\gamma$). All measurements are performed using a cone-plate configuration with a 35 mm diameter and $\alpha =$ 1° cone angle. In this configuration, $\tau$ and $\gamma$ are obtained from the measured torque, *M*(*t*), and the angular displacement, $\theta (t)$, through the standard geometrical relations $\tau (t)=3M(t)/(2\pi R^{3})$ and $\gamma (t)=\theta (t)/\alpha$, and under the standard configuration assumptions. Each hydrogel sample is freshly prepared and immediately positioned on the lower plate. The upper plate is then lowered until reaching the specified gap (0.051 mm), inducing slight pre-stress due to gel confinement. To prevent dehydration, the sample perimeter is sealed with a low-viscosity oil (0.1 Pa s).

Hydrogels polymerize in situ for 2 h at $37\,^{\circ}{}$C under cyclic strain (0.5% amplitude and 0.1 Hz), within the linear viscoelastic regime (LVE). The storage ($G'$) and loss ($G''$) modulus are continuously recorded. They represent the elastic response and energy stored during deformation, and the energy dissipation due to viscous flow, respectively. From these parameters, additional properties are derived, such as the phase angle ($\delta$), complex modulus ($G^{*}$), and dynamic viscosity ($\eta$), which is a measure of resistance to flow.

Upon completion of polymerization, the gels undergo cyclic oscillatory stress sweeps at 0.1 Hz. In stress-controlled mode, the rheometer applies the minimum torque permitted (5 ${\upmu {\text {N}}\,{\text {m}}}$), which corresponds to a minimum stress of 0.446 Pa for the given geometry. The applied stress levels generate strain amplitudes that exceed the LVE regime. Therefore, the oscillatory measurements correspond to large-amplitude oscillatory shear (LAOS) conditions, characterizing the material response in the nonlinear viscoelastic regime (NLVE).

For each imposed stress amplitude, the steady-state strain amplitude is extracted and averaged over consecutive oscillation cycles. The resulting paired values of stress and strain amplitudes are used to construct the stress–strain curves analyzed in this study. The procedure is schematically illustrated in Fig. S1 (Supplementary Material 1).

Each formulation is tested in multiple replicates. Only valid data sets are retained for analysis, requiring a minimum of two to account for the intra-material variability. Three valid tests are obtained for all formulations, except for fibrin hydrogels at 3 and 5.75 mg/mL, which include two valid tests each, and fibrin hydrogel with ADM, which includes four. This dataset forms the experimental basis for computational characterization and variability analysis. Envelope bands are constructed from the resulting curves to assess the reproducibility and consistency of the measurements.

Although full viscoelastic data are collected, $G''$ remained significantly lower than $G'$, and the maximum measured $\delta$ is below 5° (0° corresponds to purely elastic behavior). To further assess the intra-cycle response under large strain amplitudes, Lissajous–Bowditch curves are examined at $\gamma _{0}$ = 3% and $\gamma _{0}$ = 50% within the NLVE regime (Fig. S2, Supplementary Material 1). The resulting narrowly elliptical trajectories exhibit minimal enclosed area, indicating limited intra-cycle energy dissipation and a predominantly elastic response.

### Modeling Framework and Assumptions

For the computational characterization, we employ a single-phase modeling approach based on hyperelastic energy density functions, $\psi$. Hyperelastic behaviour is highly dependent on composition and microstructural organization [25]. The parameterization of this model is derived from rheological test data (“Rheological Measurements” section), ensuring an accurate representation of the mechanical properties of each material.

#### Region of Interest

Mechanical behavior to be reproduced corresponds to the stage previous of rupture initiation. To restrict the stress–strain curves to the region of interest, we develop an algorithm in MATLAB (MathWorks, Natick, MA, USA) that identifies the strain value associated with the largest drop in stiffness (G$'$) by means of a centered finite difference. Only the preceding data is retained, ensuring that the selected region remains physically consistent with the behavior to be modeled. This process corresponds to the third step in the workflow diagram, Fig. 1.

Even under fixed conditions and the same material batch, the resulting stress–strain curves are similar but not identical. This highlights the inherent variability of biological materials, known as intra-material variability, which must be considered when analysing differences between materials.

#### Preliminary Analysis

To establish an initial framework for hyperelastic characterization, Abaqus (Dassault Systemes, VlizyVillacoublay, France) *Evaluate* tool is employed [35]. This tool facilitates examining $\psi$ and computing its coefficients from experimental data, enabling the identification of the model that best captures the observed mechanical behavior. This corresponds to the fourth step in the workflow diagram, Fig. 1.

However, it is limited to a set of experimental setups, which exclude oscillatory rheological, of interest in this research. Nevertheless, we use the tool to select the Polynomial N = 2 (Eq. 1) as our hyperelastic density function.

1 $$ \psi _{\text {P}} = \sum _{i+j=1}^{N} C_{ij} ({\overline{I_{1}}} - 3)^{i} ({\overline{I_{2}}} - 3)^{j} + \sum _{i=1}^{N} \frac{1}{D_{i}} (J - 1)^{2i} $$

this function is represented as a polynomial depending on the first and second invariants of the left Cauchy–Green tensor (${\textbf{B}} = {\textbf{F}}{\textbf{F}}^{T}$), denoted by ${\bar{I}}_{1} = {tr}({\textbf{B}})$ and ${\bar{I}}_{2}=\tfrac{1}{2}(I_{1}^{2} -{\textbf{B}}_{ij}{\textbf{B}}_{ji})$. The material parameters include coefficients associated with the isochoric term, $C_{ij}$, and those related to incompressibility, $D_{i}$. Additionally, the Jacobian, *J*, is incorporated to capture volumetric effects [35–37]. For the specific case of simple shear deformation considered in this work, the definition of the deformation gradient is given by

$$ {\textbf{F}} = \begin{pmatrix} 1 &  \gamma &  0 \\ 0 &  1 &  0 \\ 0 &  0 &  1 \end{pmatrix}. $$

The selection of N = 2 is mathematically justified by the morphology of the experimental stress–strain curves, which exhibit a single inflection point. Higher-order polynomials would therefore not provide additional descriptive benefit and may unnecessarily increase model complexity. Additionally, a first initialisation of the coefficients is provided by the tool, which allowed us to set the range of the configuration search space for the Bayesian optimization process (“Bayesian Optimization: Coefficient Hydrogel Characterization” section).

#### Bayesian Optimization: Coefficient Hydrogel Characterization

To identify the coefficients of $\psi$—Polynomial N = 2—that define each experimental curve, a Bayesian optimization algorithm is implemented [38]. The coefficients to be optimized are C01, C10, C11, C02 and C20, while D1 and D2 remain equal to 0 due to the incompressibility condition [25]. A search space is defined for each coefficient.

The goal is to minimize the fitting error, defined as the Mean Squared Error (MSE) metric [39] between the computational and experimental curves,

2 $$ {\text {MSE}} = \sum _{i=1}^{n} \left( \frac{\tau _{{\text {Comp}}, i} - \tau _{{\text {Exp}}, i}}{\max (\tau _{\text {Exp}})} \right) ^{2}, $$

where $\tau _{\text {Exp}}$ and $\tau _{\text {Comp}}$ are the stress values from the experimental and computational curves, respectively *n* is the number of comparison points.

The computational curves are calculated using FE simulations for each coefficient set. For each set, a simplified FE model is built including fixed geometry, boundary conditions, meshing, and varying the selected coefficients, which define the material properties (details are provided in Supplementary Material 2, where a full FE model of the test is presented to show that the simplified model is representative of the experimental text). Under this setup, the resulting behavior only depends on the selected coefficients. To compute the MSE, each curve is compared with the experimental data from the material for which the coefficient setup is defined.

The coefficient fit search is guided by Bayesian optimization. A Gaussian-process (GP) regression model is employed as a probabilistic surrogate function of the MSE objective metric [40]. This surrogate function provides a probabilistic estimate of both the expected value and uncertainty of the objective MSE function across the coefficient search space. The prior is defined as a zero-mean GP with unit variance, and the posterior distribution is updated at each iteration using the observed MSE values.

**Fig. 2:**
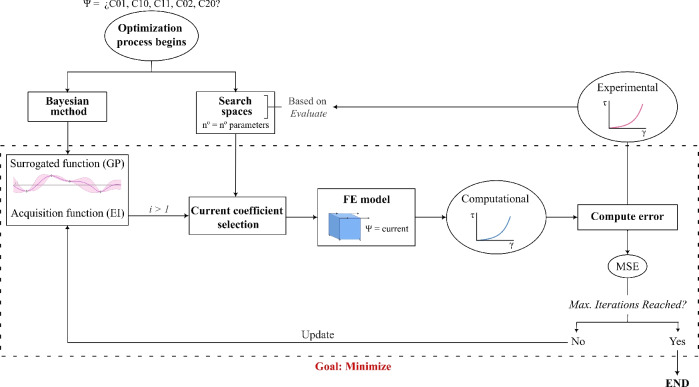
Bayesian optimization algorithm flowchart. The iterative workflow includes coefficient selection within the predefined search space, computation of the computational curve and error metric (MSE), and guidance of subsequent iterations by the Bayesian optimization algorithm

The coefficient set to evaluate in each iteration is selected by the acquisition function, Expected Improvement (EI). In the first iteration, it is random to seed the GP model. However, in subsequent iterations, the EI criterion balances exploration of regions where the GP predicts high uncertainty with exploitation of areas expected to produce low MSE. This procedure enables an efficient search for the global minimum of the objective and offers clear advantages over classical least-squares fitting [41]. The complete algorithm workflow is illustrated in Fig. 2.

The Bayesian optimization loop is implemented in Python using the Weights & Biases (W&B) library [42], which enables real-time monitoring of convergence and parameter sensitivities. Iterations continue until the convergence criteria are satisfied, resulting in the coefficient combination that minimizes the MSE and yields the closest agreement between computational and experimental stress–strain curves. This procedure is the sixth step in the workflow diagram, Fig. 1.

#### Statistical Analysis of Material Variability

Once the $\psi$ coefficients—C01, C10, C11, C02, and C20—are determined for each experimental curve, a two-step statistical analysis addresses the intrinsic variability. The first step, “Derivation of Generic Hyperelastic Function” section, focuses on intra-material variability, which is evidenced by the differences between stress–strain curves from samples of the same material tested under fixed conditions. A representative generic $\psi$ function is derived for each material, along with variability bounds defined by the extreme values of the C$_{\text {ij}}$ coefficients. The second step, “Variability of Mechanical Response as a Function of Composition” section, addresses inter-material variability by analyzing how changes in hydrogel composition affect the $\psi$ coefficients. This allows for identifying relationships between composition and mechanical response, providing insight into how material formulation influences performance. The analysis is performed in Minitab 17 [43] and represents the final step of the workflow (Fig. 1).

#### Derivation of Generic Hyperelastic Function

The objective of this part is to establish the mean and range of the $\psi$ coefficients that characterize each material. Two complementary methods were compared.

*(1) Regression model: mean curve and prediction intervals*

First approach proposes a regression model [44] to estimate the mean curve and the prediction intervals (PI) at 95%, which reflects the range of expected behavior variation. These curves are then fitted with the Bayesian algorithm to determine the coefficients. This procedure is effective but computationally expensive, requiring on average three days of calculation per material.

*(2) Coefficient estimation by statistical normalization*

The second approach is based on the assumption of coefficient normality. The coefficients obtained from individual tests are transformed to a standard normal distribution,

3 $$ \frac{{\text {C}}_{ij} - \mu ({\text {C}}_{ij})}{\sigma ({\text {C}}_{ij})} \sim {\mathcal {N}}(0, 1) $$

being $\mu$ the mean and $\sigma$ the standard deviation, non-zero due to the variability of intra-material behavior. Normality is verified using probability plots [45]. After accepting the null hypothesis, $H_{0}$, which established that the data follow a normal distribution [46], the mean values and factability intervals (FI) are calculated,

4 $$ FI = \mu (C_{ij}) \pm Z_{1 - \frac{\alpha }{2}} \cdot \sigma (C_{ij}) $$

being $Z_{1 - \frac{\alpha }{2}}$ the ${1 - \frac{\alpha }{2}}$ percentile of the standard normal distribution. These intervals define percentiles that represent extreme situations within the expected range of the material. This method reduces computation to a few hours per material.

#### Variability of Mechanical Response as a Function of Composition

Statistical methods are applied to analyze how coefficient variability depends on composition. Each group of studied hydrogels is analyzed separately.

For basal membrane extract-based hydrogels, each coefficient is treated as a response variable in a one-way ANOVA [47]. To identify significant differences between materials, post hoc comparisons are conducted using Tukey’s test [47], which is widely employed for its ability to control the Type I error rate at $\alpha = 0.1$. For fibrin-based hydrogels with liver dECM, a two-way ANOVA is used to evaluate the effects of fibrin concentration and dECM age on the coefficients. A significance level of $\alpha = 0.1$ is chosen for all analyses, considering the reduced amount of data used.

## Results

We present the computational modeling results for the different groups of hydrogels. These include the calibrations obtained using the optimization algorithm, as well as the analysis of the intra- and inter-material variability through statistical approaches.

### Bayesian Optimization Enables Consistent and Adaptive Hydrogel Characterization

**Fig. 3:**
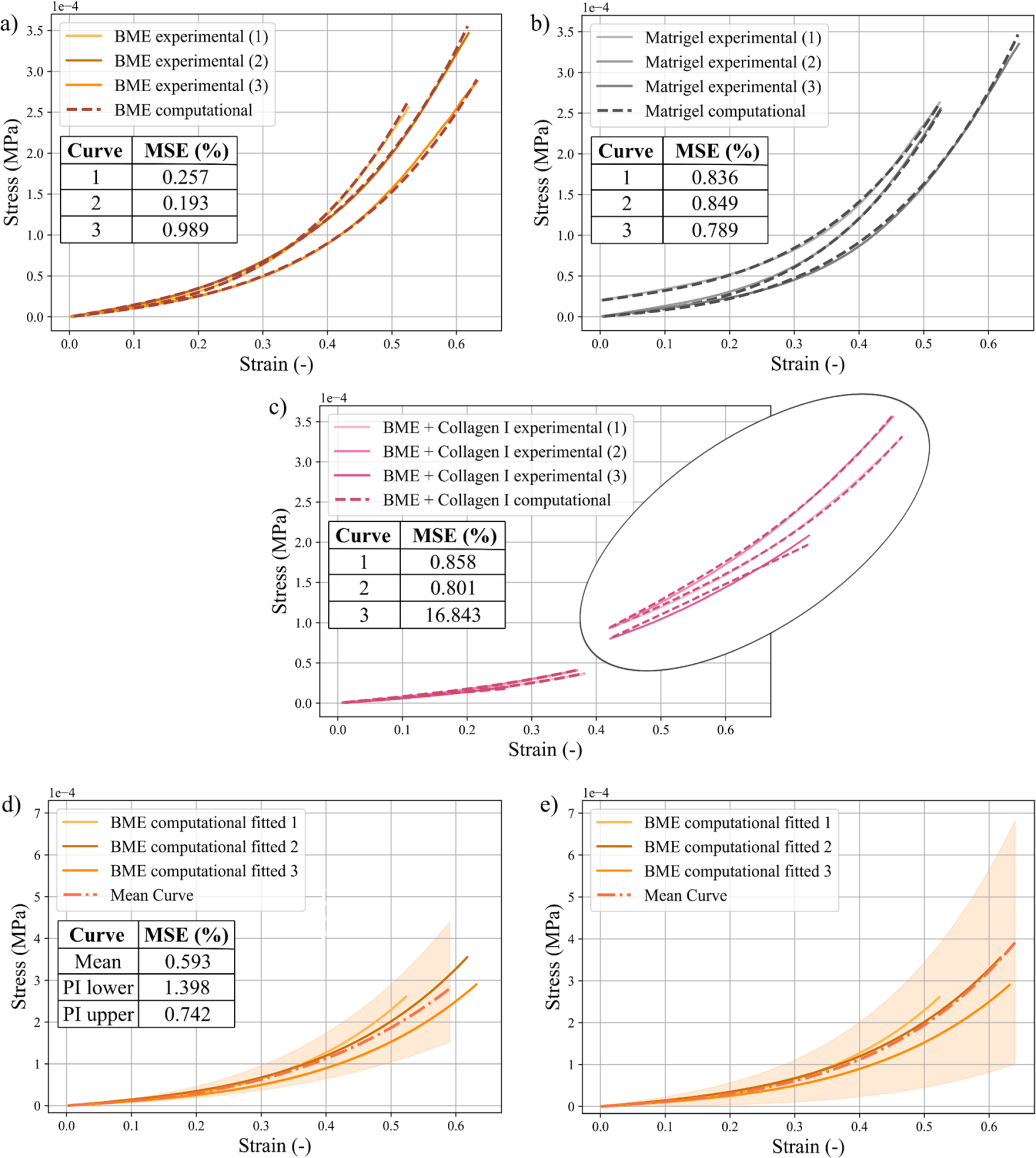
Comparison between experimental and simulated $\tau$ (MPa)–$\gamma$ (–) curves for different samples of basal membrane extract-based hydrogels. For each pair of curves there are indicated the MSE values obtained using the optimization algorithm, indicating the quality of the fit. All plots are shown at the same scale; zoomed-in insets are provided in plots where the fitting details are unclear. There are shown the following materials: **a** BME, **b** Matrigel (with an offset of $2.00 \times 10^{-5}$ for the first curve set), and **c** BME with Collagen Type I (with an offset of $-2.00 \times 10^{-6}$ units for the third). Offsets are applied for improved visualization. **d** and **e** Display the results of the statistical analysis conducted to generalize the mechanical behavior of BME, “Derivation of Generic Hyperelastic Function” section. Both plots show the mean $\tau$–$\gamma$ curve along with the variability range given by the individual material responses. **d** Corresponds to the regression-based approach, including MSE values. **e** Shows the results from the statistical normalization method. The shaded areas represent the 95% PIs in **d**, and the 80% FIs in **e**

**Fig. 4:**
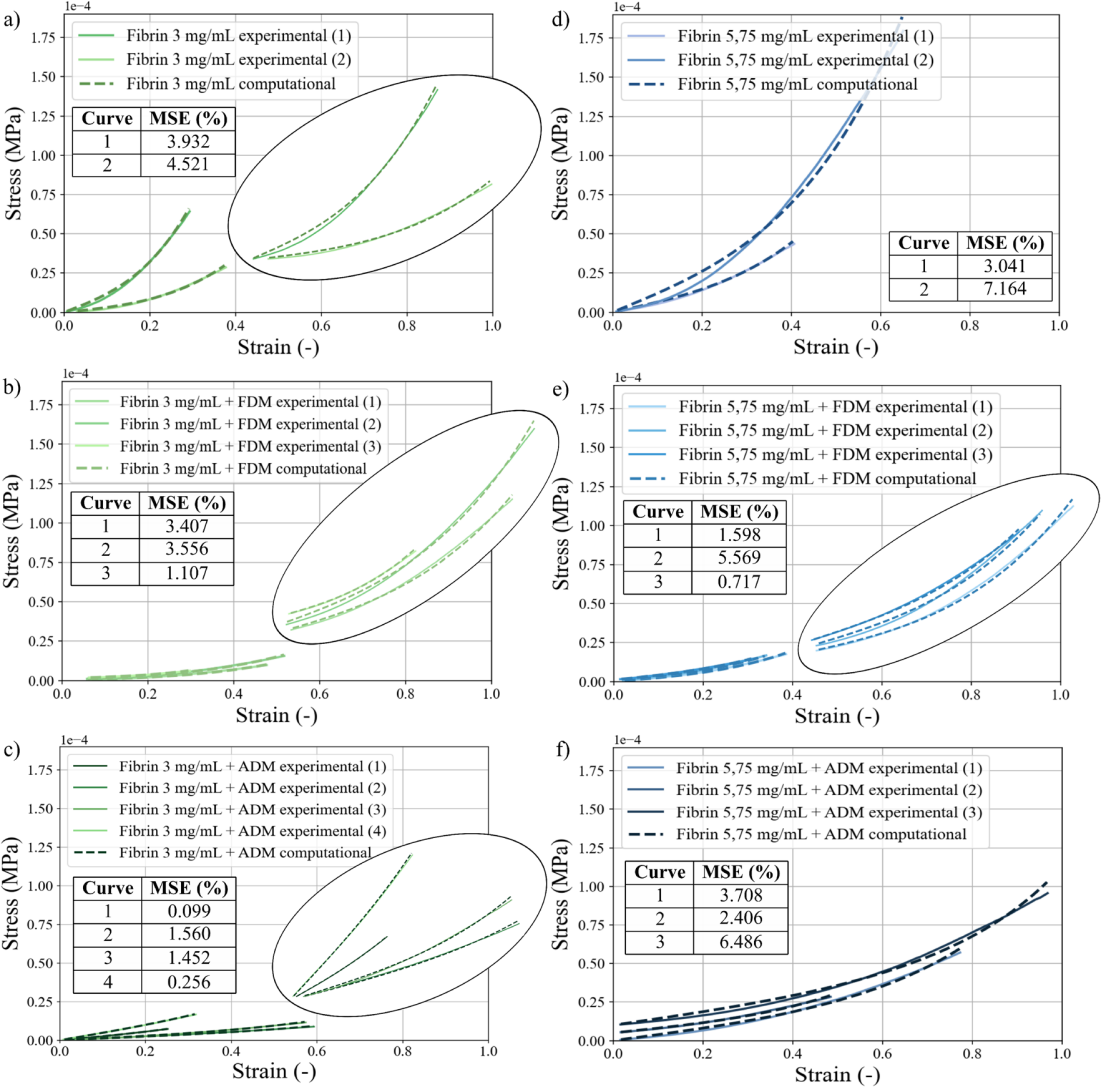
Comparison between experimental and simulated $\tau$ (MPa)–$\gamma$ (–) curves for different samples of fibrin-based hydrogels with or without decellularized liver matrix. For each pair of curves, the MSE values obtained from the optimization algorithm are indicated, reflecting the quality of the fit. All plots are shown at the same scale; zoomed-in insets are provided in plots where the fitting details are unclear. Subfigures **a**–**c** Correspond to hydrogels with a fibrin concentration of 3 mg/mL—shown in shades of green—without decellularized matrix, with ADM and FDM (offsets of $5.00 \times 10^{-7}$ and $1.40 \times 10^{-6}$ units for the second and third sets), respectively. Subfigures **d**–**f** show 5.75 mg/mL fibrin hydrogels with the same dECM compositions—shown in shades of blue. Offsets were also applied to ADM ($5.00 \times 10^{-6}$ and $1.00 \times 10^{-5}$ for the second and third sets) and FDM ($5.00 \times 10^{-7}$ and $1.00 \times 10^{-6}$, respectively). Offsets are applied for better visualization

The computational fits obtained through the Bayesian optimization algorithm from experimental data are shown in Figs. 3a–c and 4, corresponding to basal membrane extract-based and fibrin-based hydrogels, respectively. Each pair of experimental and simulated stress–strain curves is accompanied by the corresponding MSE value, which allows identification of the best fit. In all cases, low MSE values confirm that the experimental curves are accurately reproduced by the selected $\psi$ function.

For basal membrane extract hydrogels, Fig. 3a–c, all MSE values remain below 1%, except for the third replicate of BME combined with collagen type I.

For fibrin-based hydrogels, Fig. 4, curves are color-coded by fibrin concentration, green for 3 mg/mL and blue for 5.75 mg/mL. The algorithm consistently achieves low and stable MSE values below 7%, evidencing both robustness and adaptability across different formulations.

The fitted hyperelastic coefficients—C01, C10, C11, C02, C20—for each sample and material are reported in Supplementary Material 3, Tables S1 and S2.

### Intra-material Mechanical Variability Explored by Statistical Modeling

To characterize intra-material variability, a statistical analysis is employed (“Derivation of Generic Hyperelastic Function” section) to define each material as a representative general expression with variability ranges that capture behavioral diversity while maintaining physically coherent limits. A dual methodological approach is implemented depending on the statistical assumptions satisfied by each dataset; Table 1 summarizes it for each hydrogel.

Both approaches are applied to BME for comparison. Regression model reveals a statistically significant cubic relationship between $\tau$ and $\gamma$. From this, a mean curve and 95% PIs are defined, Fig. 3d. This is extrapolated to represent the general mechanical behavior of the hydrogel. In parallel, a coefficient-based approach is used defining an 80% FI, Fig. 3e. Although more manual and less robust, this approach offers a lower computational cost and captures a broader variability range. As shown, both analyses are complementary.

Coefficient-based methodology is applied to the remaining basal membrane extract hydrogels, with p-values $>0.1$ confirming normality. FIs levels are set at 90% for Matrigel and 60% for BME with collagen type I, ensuring physically consistent mechanical bounds.

For fibrin-based hydrogels, the methodology is selected case by case. In formulations without dECM—fibrin at 3 mg/mL and 5.75 mg/mL—normality assumptions are not met, prompting the use of a second-degree regression model to capture nonlinear $\tau$–$\gamma$ relationships. Conversely, coefficient-based analysis is applicable for hydrogels containing dECM. FI levels are set at 75% for 3 mg/mL formulations, and at 80% and 70% for 5.75 mg/mL ADM and FDM hydrogels, respectively. These thresholds are selected to provide the widest physically consistent intervals in the lower bounds of mechanical response.

**Table 1:** Summary of the statistical approaches implemented to address intra-material variability across hydrogel formulations

Method	Basal membrane extract	FI	Fibrin w/ dECM	FI
Regression model	BME	–	3 and 5.75 mg/mL	–
Coefficient normality	BME	80%	3 mg/mL ADM/FDM	75%
	Matrigel	90%	5.75 mg/mL ADM	80%
	BME w/ collagen type I	60%	5.75 mg/mL FDM	70%

The table specifies the applied method and the corresponding FI for the coefficient normality-based analysis

The mean and variability coefficients obtained through statistical approaches are reported in Supplementary Material 3, Tables S3 and S4.

### Basal Membrane Extract-Based Hydrogels Behaviour is Modulated by Collagen Type I Incorporation

This section examines how the composition of basal membrane extract-based hydrogels influences their mechanical response (“Statistical Analysis of Material Variability” section) by analyzing the fitted $\psi$ coefficients.

To interpret the mechanical meaning of each coefficient, a sensitivity analysis is conducted in Abaqus by systematically varying each one individually while keeping the others fixed. The analysis reveals that C01 and C10 primarily influence the initial slope, the low-strain region. An increase in these parameters indicates higher initial stiffness. C11 governs the intermediate region and is directly associated with the material’s non-linearity, with higher values resulting in steeper curves. Finally, C02 and C20 affect the final slope, associated with the high-strain region. An increase in values corresponds to increased stiffness under large deformations. A representation of the resulting variations in curve morphology, associated with individual coefficient modulation, is illustrated in Supplementary Material 3 Fig. S5. This approach enables interpreting subsequent statistical comparisons rather than purely symbolic ones.

The main ANOVA results for this group of materials are presented in Table 2. C01, C10, and C20 show no significant variation across formulations, p-values > 0.1, suggesting comparable behavior for these parameters. In contrast, p-values of C11 and C02 reveals meaningful differences, indicating that composition-dependent changes primarily affect nonlinearity and stiffness at large deformations.

Except for C02, adjusted $R^{2}$ values are relatively low, suggesting limited explanatory power of the models in capturing variability based on formulation. Notably, high F-values tend to correspond with low p-values and vice versa, reinforcing the strength or absence of association between specific coefficients and hydrogel composition.

**Table 2:** Summary of one-way ANOVA results for the model coefficients, $\psi$, across the basal membrane extract-based hydrogels

Coefficient	C01	C10	C11	C02	C20
$R^{2}$***-adjusted***	20.27%	14.52%	38.95%	97.55%	32.23%
***F-value***	2.02	1.68	3.55	160.11	2.90
***p-value***	0.214	0.263	0.096	0.000	0.131

The adjusted $R^{2}$ values indicate the proportion of variance explained by the model, while F-values and p-values assess the statistical significance of differences among group means

ANOVA results identify C11 and C02 as the primary descriptors of mechanical differences. The following analyses examine these coefficients in detail between formulations.

#### BME and Matrigel Exhibit a Similar Non-linear Behaviour

To further explore the role of hydrogel composition in mechanical performance, we first compare the two standard formulations: BME and Matrigel.

ANOVA and Tukey’s post hoc test of the C11 coefficient are presented in Table 3. They indicate no statistically significant difference, as reflected by overlapping CI and grouping labels, although Matrigel shows a higher mean value. Both formulations therefore exhibit comparable non-linearity in the intermediate strain range.

In contrast, the analysis of the C02 coefficient presented in Table 3 reveals significant differences between BME and Matrigel, with BME exhibiting the highest mean value. However, this variation does not translate into a clear difference in macroscopic behavior. Matrigel presents the highest mean for C20, although without statistical significance, and as shown Fig. 3a, b both hydrogels reach similar stress and strain levels under mechanical loading and exhibit similar mechanical behaviour up to failure. Given that C02 and C20 contribute equal to the response at high strain, it is reasonable that their combined effect appears to balance the overall behaviour of the two materials.

Finally, as also illustrated in Fig. 3a, b, both BME and Matrigel exhibit similar mechanical behaviour. Despite inherent variability, they reach comparable stress and strain levels prior to rupture, suggesting they can withstand equivalent mechanical demands.

**Table 3:** ANOVA coefficients and Tukey’s post hoc test results for C11 and C02

Coeff.	Material	Mean	90% CI	Grouping
C11	BME	0.000049	(− 0.000020; 0.00012)	A B
	Matrigel	0.000144	(0.000076; 0.000213)	A
	BME w/ Collagen type I	0.000016	(− 0.000053; 0.000085)	B

Coeff.	Material	Mean	90% CI	Grouping
C02	BME	0.00016	(0.000146; 0.000172)	A
	Matrigel	0.000025	(0.000012; 0.000038)	B
	BME w/ Collagen type I	0.000007	(− 0.000006; 0.000020)	B

The table shows the mean values, 90% CI and groupings for each basal membrane extract-based hydrogels. Materials sharing the same letter are not significantly different from each other at the 90% confidence level

#### Collagen Type I Reduces Non-linearity of the Mechanical Behaviour

To evaluate the mechanical impact of include collagen type I on basal membrane extract-based hydrogels, we compare it with the standard formulations.

As shown in Table 3, ANOVA followed by Tukey’s post hoc test reveal that Matrigel and BME with collagen type I are significantly different in terms of non-lineality, C11. They represent the extreme curvature situations, reflected in the mechanical response curves shown in Fig. 3b, c, where the addition of collagen type I clearly reduces non-linearity, yielding a more linear behaviour.

Moreover, the addition of collagen type I decreases mechanical resistance and deformation capacity, reaching the failure at lower stress and strain levels in comparison with BME and Matrigel, Fig. 3a–c. These results indicate a limited capacity to withstand mechanical loads when compared to the standard formulations.

### Fibrin Concentration and dECM Age Control Hydrogel Mechanical Behaviour

To investigate the mechanical effects of fibrin concentration and dECM age on fibrin-based hydrogels, an ANOVA test is performed (“Variability of Mechanical Response as a Function of Composition” section).

These two variables allow to formulate initial hypotheses of its effect in the expected variation of the model $\psi$ coefficients, that define the mechanical response. This rationale is based on prior knowledge and informed by sensitivity analysis, “Basal Membrane Extract-Based Hydrogels Behaviour is Modulated by Collagen Type I Incorporation” section. We hypothesise that C01, C10, C02, and C20 increase with both fibrin concentration and dECM age, reflecting the progressive stiffening of biological tissues. As noted by Wood et al., these factors are interconnected through the aging process [48], supporting this hypothesis. In contrast, the C11 is expected to decrease as fibrin concentration and dECM age increase, since greater stiffness is generally associated with a more linear mechanical response.

#### Mechanical Stiffness at Low Strains Increases with Fibrin Concentration and dECM Age

Main ANOVA results for C01 and C10 are summarised in Tables 4 and 5.

Both fibrin concentration and dECM age significantly influence the coefficient C01, Table 4, with dECM age showing the strongest influence (p-value $\ll$ $\alpha$). The sign and magnitude of the estimated model coefficients is relevant in the understanding of these effects, Table 5. Fibrin concentration shows a statistically positive relevant influence on C01, p-value < $\alpha$ and also the incorporation of adult dECM. In contrast, fetal dECM exhibit a negative coefficient with a non-significant p-value of 0.577. These results indicate that mature dECM effectively enhances the mechanical stiffness associated with C01, while fetal dECM has a limited influence.

When factors are considered in combination, both increasing fibrin concentration or dECM age result in elevated C01 values. The stiffest mechanical response is observed in hydrogels containing adult dECM, followed by those with fetal dECM and is lowest in formulations without any dECM.

For C10, results reveal a statistically significant influence of both fibrin concentration and dECM age, supported by their p-values in Table 4. Fibrin concentration is associated with a positive coefficient, reflected in Table 5, suggesting again that increasing it leads to greater initial stiffness. In contrast, both adult and fetal dECM groups present significant negative coefficients, more pronounced in the fetal group. The absence of dECM yields the highest C10 values, although this increase is not statistically significant.

Together, these results indicate that both increasing fibrin concentration and dECM age contribute to the enhancement of C10, although the most notable increase occurs in the absence of dECM but without statistically significance.

**Table 4:** ANOVA results for the fitted coefficients C01 and C10 based on the influence of fibrin concentration and dECM age

Variable	Coeff	F-value	p-value	Coeff	F-value	p-value
Concentration		3.89	0.072		4.18	0.063
Age	C01	5.53	0.020	C10	12.64	0.001
$R^{2}$-adjust		45.05%			66.84%	

ANOVA results for the fitted coefficients C01 and C10 based on the influence of fibrin concentration and dECM age. Fvalues and p-values are reported for each independent variable and $R^{2}$ - adjusted values (45.05% for C01 coefficient and 66.84% for C10 coefficient) indicate the explanatory power of the models for each coefficient

**Table 5:** Estimated model coefficients of the ANOVA analysis for C01 and C10, and associated p-values, derived from the effects of fibrin concentration and dECM age

Term	Coeff	ANOVA coef	p-value	Coeff	ANOVA coef	p-value
Concentration		$1.00 \times 10^{-6}$	0.072		$2.00 \times 10^{-6}$	0.063
Adult	C01	$4.00 \times 10^{-6}$	0.006	C10	$-4.00 \times 10^{-6}$	0.036
Fetal		$-1.00 \times 10^{-6}$	0.577		$-6.00 \times 10^{-6}$	0.003

Positive and negative signs of the coefficients reflect the direction of the factor effect on each mechanical parameter

These findings are consistent with the hypothesis originally formulated and supported by Wood et al. [48]. Tissue stiffness increases with both biological age and matrix density, analogous to the increase in fibrin concentration.

#### Reduction of Nonlinear Elasticity with Increasing dECM Age

The ANOVA analysis of the C11 parameter is presented in Table 6.

The parameters reveal that fibrin concentration exhibits a marginal effect, p-value of 0.124, slightly exceeding the significance threshold adopted. However, it presents a positive coefficient, suggesting a modest increase in C11. In contrast, dECM age exerts a statistically significant influence as indicated by its null p-value. Furthermore, the inclusion of adult dECM results in a significant negative relation with C11. A similar trend is observed with fetal dECM, although less pronounced.

**Table 6:** Main ANOVA results and estimated model coefficients for the C11 parameter

Variable	Coeff	F-value	p-value	Term	ANOVA coef	p-value
Concentration	C11	2.78	0.124	Concentration	$3.00 \times 10^{-6}$	0.124
Age		33.76	0.000	Adult	$-2.10 \times 10^{-5}$	0.000
				Fetal	$-1.00 \times 10^{-5}$	0.006

Main ANOVA results and estimated model coefficients for the C11 parameter. F-values and p-values from the ANOVA analysis and R2-adjusted value (83.07%) assess the influence of fibrin concentration and dECM age on the mechanical parameter C11. Estimated model coefficients and their associated p-values are also provided, revealing the direction and statistical relevance of each factor’s effect on nonlinear elasticity

The findings confirm that increasing dECM age is associated with a reduction in the C11 coefficient. This aligns with the initial hypothesis proposed and supported by Wood et al. [48], which links tissue aging to a reduction in nonlinear elasticity.

#### Equivalent Mechanical Behavior via Compensation Between Fibrin Concentration and dECM Age at Low and Middle Strains

An important outcome of the analysis of C01, C10, and C11 is the identification of a compensatory relationship between fibrin concentration and dECM age in modulating mechanical behaviour. Specifically, increasing fibrin concentration in hydrogels containing younger dECM can reproduce the mechanical effects observed with older dECM at lower concentrations, and vice versa. This finding enables the design of hydrogels with comparable mechanical properties through different combinations of these two factors. Such versatility is valuable in hydrogel formulation, allowing for precise tuning of mechanical performance to meet specific application requirements, while also contributing to more efficient use of materials.

#### Mechanical Response at Large Strains is Independent of Fibrin Concentration and dECM Age

ANOVA analysis for C02 and C20 are presented in Table 7. The results indicate that neither fibrin concentration nor dECM age exerts a statistically significant effect on these coefficients, as reflected by their p-values. Furthermore, the estimated ANOVA models yield low $R^{2}$ and F-values, indicating poor model fit. Consequently, it can be concluded that the mechanical response at large strains, characterized by C02 and C20, is not significantly modulated by changes in fibrin concentration or dECM age.

**Table 7:** Main ANOVA results for coefficients C02 and C20 of fibrin-based hydrogels

Variable	Coeff	F-value	p-value	Coeff	F-value	p-value
Concentration	C02	0.17	0.692	C20	0.81	0.383
Age		1.66	0.231		2.34	0.136

Main ANOVA results for coefficients C02 and C20 of fibrin-based hydrogels. F-values, p-values and $R^{2}$-adjusted values (22.04%% for C02 coefficient and 12.86% for C20 coefficient) indicate the absence of statistically significant effects and a poor model fit for both coefficients

#### Adding dECM Reduces Stiffness of Fibrin-Based Hydrogels

A general analysis of the mechanical behavior of fibrin-based hydrogels is provided, with stress–strain curves shown in Fig. 4.

In the subgroup of hydrogels without dECM (Fig. 4a, b), the formulation with higher fibrin concentration reaches greater stress levels before failure. This indicates a more resilient response under increased mechanical load. When dECM is added, this effect is maintained.

FDM formulations (Fig. 4c, d), lead to notably lower stress levels prior to failure, compared with the corresponding hydrogels without dECM. This indicates reduced mechanical strength, although deformability remains unaffected.

In ADM formulations (Fig. 4e, f), the effect is more pronounced with lower fibrin concentration. The maximum stress values are considerably reduced compared to their respective formulation without dECM (Fig. 4a, b), while increasing the deformation level. In the case of higher fibrin concentration, stress values are also reduced with respect to its dECM formulation, although to a lesser extent. Overall, ADM appears to reduce the mechanical strength of the materials while increasing their deformability.

Comparing the FDM and ADM formulations (Fig. 4c–f), those containing ADM exhibit greater stiffness, as indicated by a steeper initial slope, and reach higher levels of deformation. This suggests that although both types of dECM reduce mechanical strength, ADM confers a greater capacity for deformation.

## Discussion

This study establishes an integrated experimental–computational methodology for the quantitative characterization of next-generation hydrogels specifically designed for TE. The experimental stage involves two main parts. First, the controlled synthesis of custom hydrogel formulations is achieved by varying key parameters such as concentration or the main biological source. Then, their mechanical characterization was performed through oscillatory rheological tests under controlled conditions. On the computational side, the framework integrates a Bayesian optimization algorithm for robust hyperelastic coefficient identification, complemented by statistical methods to capture intra- and inter-material variability. The systematic integration represents a relevant contribution that has not been previously reported. It enables an accurate constitutive description of hydrogel mechanics, while explicitly quantifying the inherent heterogeneity of biological materials [49, 50], whose microstructural nature is highly variable even in the same batch.

The Bayesian optimization algorithm provides a consistent and adaptive strategy to characterize the mechanical behavior of the hydrogels. Its iterative refinement enabled robust coefficient fitting [51], finally adjusting the hyperparameters to the observed response. While this machine learning strategy has been widely adopted in biomedical domains such as patient-specific mechanical characterization, exemplified by Ross et al. [52], and constitutive modeling or design optimization, such as Ghoreishi et al. [53], its use here highlights clear advantages over traditional fitting methods, which typically show slower convergence and higher sensitive to local minima.

Applied to the hydrogels tested in this study, the method yielded generally low fitting errors. The highest discrepancies were observed in materials with more complex compositions, such as liver-derived dECM. An exception was noted in the third repetition of BME with collagen type I hydrogel, potentially due to inconsistencies in the experimental data. Nevertheless, the algorithm adapted well to morphological differences across samples, consistently achieving accurate fits. Overall, the selected model, guided by the Bayesian optimization, proved flexible and capable of capturing the underlying physics of protein composition and microstructural organization in the material’s response. However, a hierarchical Bayesian framework [54] would be a rigorous data treatment by explicitly modeling correlations between hydrogels and repetitions, and will be considered in future work.

The intra-material variability, well-recognized in biological systems, is explored by statistical modeling. This approach enabled us to parametrize the deviation in the mechanical behavior for each hydrogel studied and to generate intervals for the constitutive parameters with a high degree of certainty, while ensuring that the inferred models preserve the physical coherence of the material behavior. By explicitly incorporating this variability, we provide a more realistic representation of hydrogel mechanics.

To complete the analysis of the overall behavior of the hydrogels, a statistical approach was used to evaluate how composition modulates mechanical behavior through the fitted constitutive parameters. Within the group of basal membrane extract-based hydrogels, the commercial products BME and Matrigel exhibit comparable non-linear behavior, as evidenced by the absence of statistically significant differences in their constitutive parameters and by the similarity of their stress–strain curves. This suggests that their compositions are not different enough to generate distinct mechanical responses, in agreement with some studies that consider both products to have essentially the same composition, differing only by commercial source, as Benton and colleagues [55].

Building on this, we investigate the effect of compositional modifications, in particular by the addition of collagen type I. The nonlinearity parameter (C11) is statistically reduced, bringing the material’s behavior closer to that of a linear elastic solid. Moreover, both stiffness and deformability of the initial material decrease, meaning the resulting composite resists lower levels of stress before failure. These observations are consistent with the study of Buchmann et al. [56], who observed a reduction in mechanical nonlinearity of Matrigel–collagen type I hybrid hydrogels. They attribute this to the suppression of collagen polymerization in the presence of Matrigel, which leads to shorter fiber lengths and a modified network structure. This microstructural change results in a less continuous and mechanically weaker network, supporting the reduced deformability and earlier fracture observed in our results. Furthermore, while Buchmann et al. used a collagen concentration of 1.3 mg/mL, our hydrogels contain a higher concentration (2.5 mg/mL), which may cause the mechanical response to be dominated by the more fragile and plastic behavior of the collagen network. Interestingly, these alterations in matrix composition have also been shown to influence cellular behavior. For instance, Anguiano et al. [57] studied how cancer cell migration is modulated by the mechanical characteristics of these hybrid hydrogels.

In the fibrin-based hydrogel formulations, we quantify the effects of fibrin concentration and dECM age on the modulation of mechanical properties. The results aligned well with our previous hypotheses and literature, which highlights how increasing concentration—closely related to fiber density—enhances matrix stiffness in the mechanical response. This occurs because denser networks restrict fiber rearrangement and deformation. Tissue age also plays a significant role. It is well established that adult tissues lose fluidity and fiber elasticity due to increased cross-linking [48] and that collagen content tends to increase with age, becoming more densely packed and organized [48] which shifts the mechanical response toward a stiffer behavior. Thus, a strong connection exists between aging and concentration. In this study, this is reflected in the observed increase in initial stiffness (C01 and C10) and the reduction in nonlinearity (C11). However, the high-strain region (C02 and C20) did not exhibit a statistically significant dependence on either concentration or dECM age. Instead, the stress levels reached at large deformations appear to be primarily influenced by the material’s preceding mechanical behavior.

These analyses inherently account for the intrinsic variability of biological materials, as they include the complete computational dataset for each formulation, capturing the diversity of responses observed across replicates.

An important finding is the mechanical tunability of fibrin-based hydrogels. Specifically, it was found that equivalent mechanical responses at low to moderate strain levels—which correspond to physiologically relevant deformation ranges—can be achieved by balancing fibrin concentration and dECM age. For example, a hydrogel with low fibrin concentration combined with older dECM age can exhibit a similar mechanical behavior to one with high fibrin concentration and younger dECM, within these deformation ranges. This compensatory effect highlights that multiple formulation strategies can yield comparable mechanical performance, offering flexibility in hydrogel design. Such tunability expands the versatility of these materials for adapting to specific biomedical applications.

From a mechanical perspective, the incorporation of dECM reduces hydrogel stiffness, indicating lower mechanical resistance to deformation. This effect is likely associated with structural alterations induced by the decellularization process, which has been shown to compromise the mechanical integrity of native tissues by removing cellular components and disrupting the ECM architecture [58]. For instance, Rukshika et al. [59] reported that including human dECM in hybrid hydrogel systems significantly reduces the average shear modulus. Nevertheless, also highlights biological advantages, such as the modulation of cellular responses and hydrogel architecture. In addition, formulations that incorporate adult-derived dECM appears to increase tissue deformability. This is consistent with previous studies reporting age-related changes in native tissues, which exhibit increased compliance and extensibility [60].

This study aligns with the growing interest in understanding how matrix mechanics modulate cellular behavior [61–63], affecting both physiological or pathological processes. For example, mechanical stress activates the TGF$\beta$ signaling pathway in multiple cell types, including valvular interstitial cells (VICs), where abnormal mechanical response can promote profibrotic phenotypes involved in myxomatous mitral valve disease [64]. Similarly, substrate stiffness directs mesenchymal stem cell (MSC) differentiation toward specific lineages, highlighting the role of mechanical cues in guiding cell fate [64]. This principle has inspired applications in vascular regenerative medicine, where it is hypothesized that biomechanical cues can steer MSCs toward smooth muscle or endothelial phenotypes [65]. More broadly, substrate mechanics influence a wide range of cellular functions, including cell–cell interactions, ion transport, and responses to interstitial flow [66]. In vivo, this complexity is further increased by the continuous remodeling of the ECM, which alters local mechanical conditions and has significant consequences for tissue homeostasis and disease progression.

When tissue mechanical balance is disrupted, it can contribute to the onset and progression of various pathological conditions, including cardiovascular diseases, fibrosis and cancer. In the central nervous system, even subtle alterations in extracellular fluid pressure or ECM stiffness can disturb the brain mechanical homeostasis, potentially impairing cognitive functions and contributing to the development of neurodegenerative diseases such as Alzheimer’s and Parkinson’s [67]. In cancer, progressive stiffening of the ECM—often driven by increased collagen deposition and fiber linearization—has been shown to facilitate tumor invasion and metastasis, as reported in breast cancer [68]. Moreover, the ability of malignant cells to withstand shear stress appears to be a conserved biophysical trait, suggesting adaptive mechanical mechanisms that support their survival and dissemination [69].

Therefore, given the rise of TE as an alternative to traditional prostheses [70], there is a pressing need to establish clear relationships between cellular behavior and substrate mechanics. Doing so, will not only enhance our understanding of numerous biological processes whose underlying mechanisms remain unclear, but also allow us to better predict cellular behavior and the bioactivity of biomaterials intended for damaged tissue replacement.

In this context, we focused on hydrogels as representative ECM-like substrates, aiming to quantify their mechanical properties. Based on our analysis and supported by the mechanobiology literature, we consider that parameters governing the initial stiffness of the material (C01 and C10), as well as those controlling its non-linear mechanical response (C11), are the most relevant for guiding cellular behavior. These parameters are expected to play a crucial role in generating mechanically mimetic environments that more accurately reproduce the conditions encountered by cells in vivo. In particular, the initial stiffness of the matrix has been widely associated with processes such as durotaxis and cell migration [71], while non-linear mechanical behavior is increasingly recognized as a critical feature of soft biological tissues [72].

Experimentally, the hydrogels exhibit a predominantly nonlinear elastic behavior, while rheological measurements also reveal a secondary viscous contribution. This component, although not dominant, is evidenced by the values of $G''$ and $\delta$, which remained significantly lower than the corresponding elastic contribution. $\delta$ showed values around 4°, which, according to the literature [73], indicates that the deformation response occurs almost instantaneously upon stress application. Having established that the intra-cycle dissipation remains limited—as evidenced by the Lissajous–Bowditch analysis—and considering the pronounced nonlinear elastic response observed in the stress–strain curves, a hyperelastic framework provides an adequate first-order mechanical description of the material behavior. A viscoelastic formulation could be developed in future works through the definition of a Prony series based on the generalized Maxwell model [32, 74].

## Conclusion

This study establishes a comprehensive and transferable methodology for the mechanical characterization of biologically derived hydrogels increasingly used in TE. The incorporation of a Bayesian optimization algorithm represents a significant advance over conventional fitting techniques, allowing accurate fitting of hyperelastic constitutive models from rheological test data providing the parameters for developing a computational simulation model. The algorithm can be applied to any material with a parameterizable mechanical response, simply by updating the constitutive model, and any type of experimental test. This extensive applicability facilitates its utilization across a broad spectrum of biomedical applications.

A salient feature of the proposed methodology is its capacity to accommodate intra-material variability of each biomaterial. This enables the definition of materials by their mechanical performance, as well as by the robustness and reproducibility of their global behaviour.

The proposed workflow has been successfully applied to characterize novel hydrogel compositions, including fibrin-based scaffolds with liver dECM and basal membrane extract-based hydrogels with added collagen type I. The methodology captures how compositional factors—such as collagen addition, fibrin concentration and tissue age—affect mechanical behavior, including stiffness, deformability and nonlinearity degree. These findings reveal relationships between composition and mechanical behavior. This understanding allows for the quantification of individual factors, enabling the optimization of biomaterial design and fabrication for specific biomedical applications.

Taken together, this methodology offers a powerful computational framework that not only advances the mechanical understanding of hydrogels but also accelerates their development and application for biomedical applications.

## Supplementary Information

Below is the link to the electronic supplementary material.

(TEX 27 kb)

(PNG 343 kb)

(PNG 543 kb)

(PNG 883 kb)

(PNG 21060 kb)

(PNG 1656 kb)

## Data Availability

The data and code developed throughout this document will be made available on request. Not applicable.
