# Supplementary figures and images for: Non-linear Characterization of Commercial and Decellularized Hydrogels: Statistical Framework Enhanced by Bayesian Optimization
Source: Cell Mol Bioeng. 2026 Jun 30;19(3):327–44. doi: 10.1007/s12195-026-00913-1 (PMC13365100; doi:10.1007/s12195-026-00913-1)

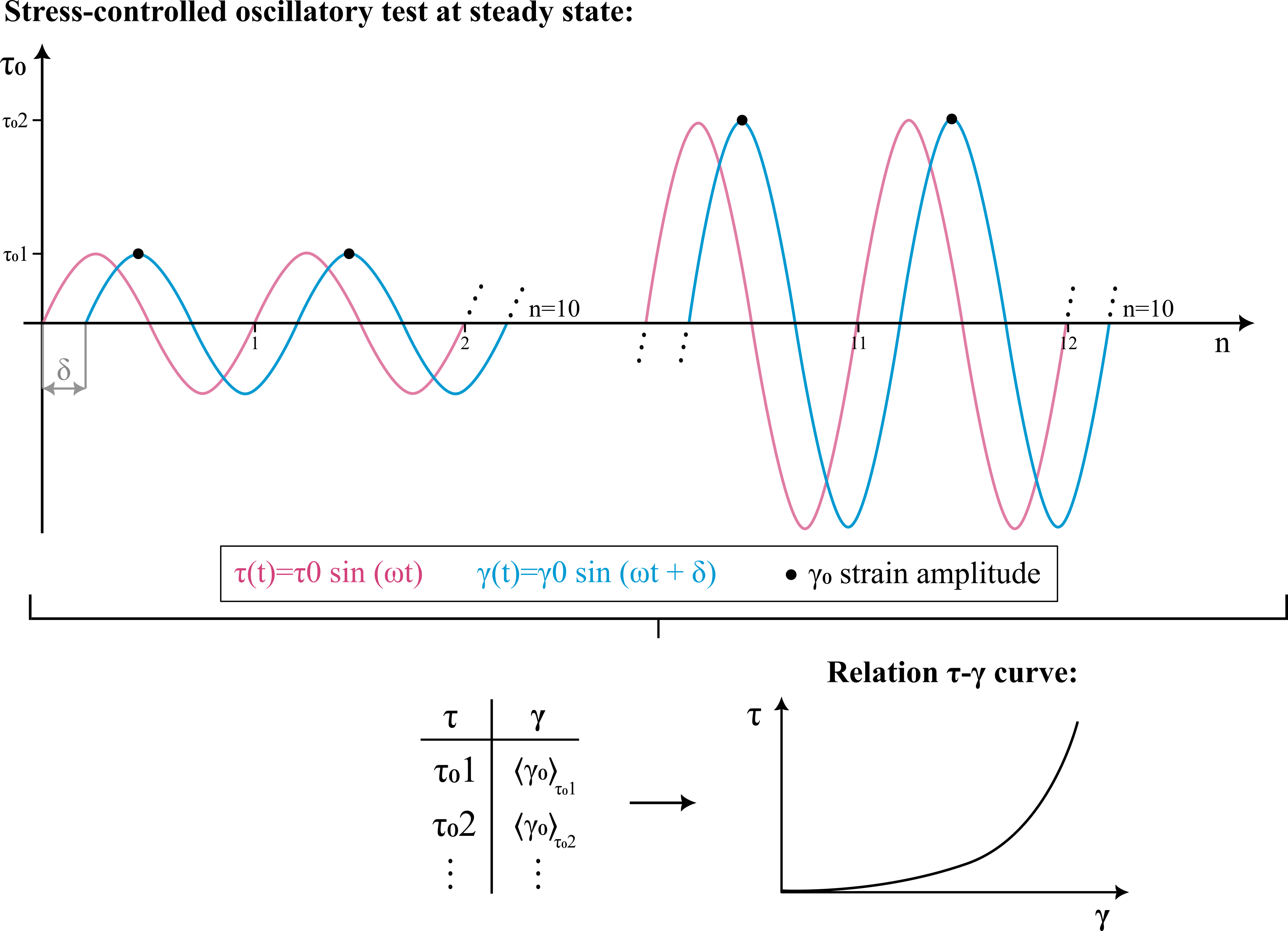

Supplement: Supplementary file 2 — (PNG 343 kb) [file 12195_2026_913_MOESM2_ESM.png]

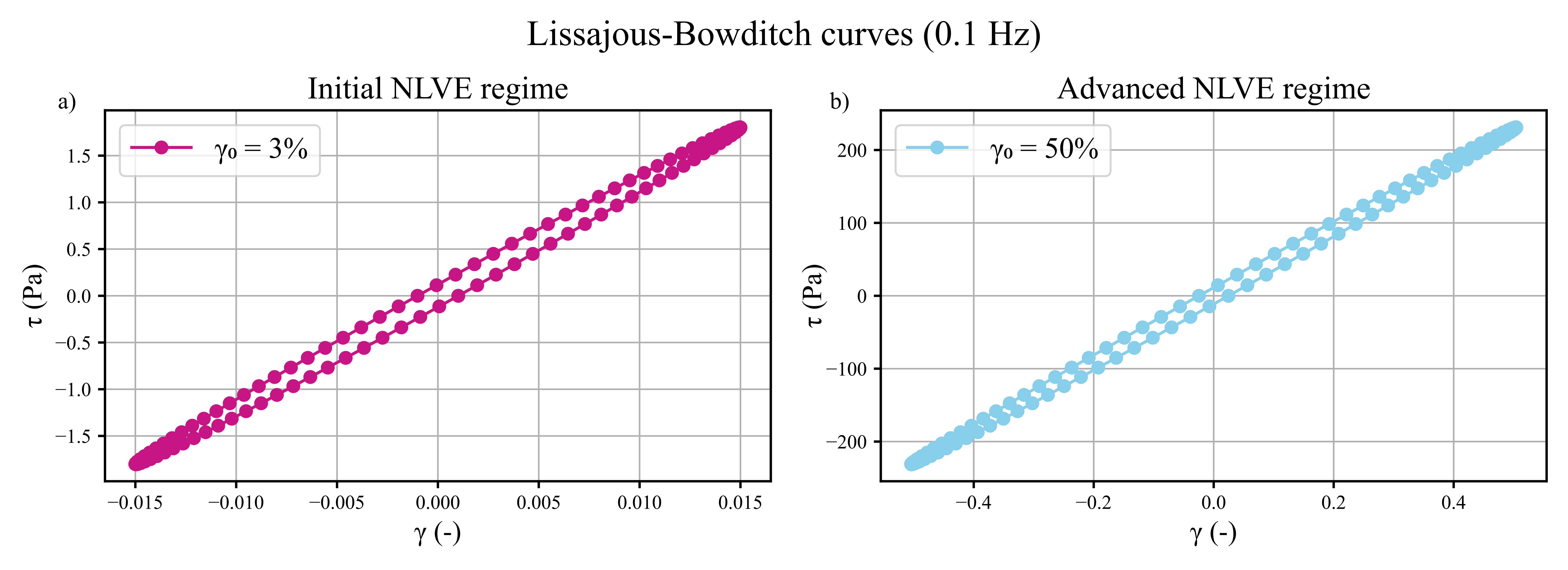

Supplement: Supplementary file 3 — (PNG 543 kb) [file 12195_2026_913_MOESM3_ESM.png]

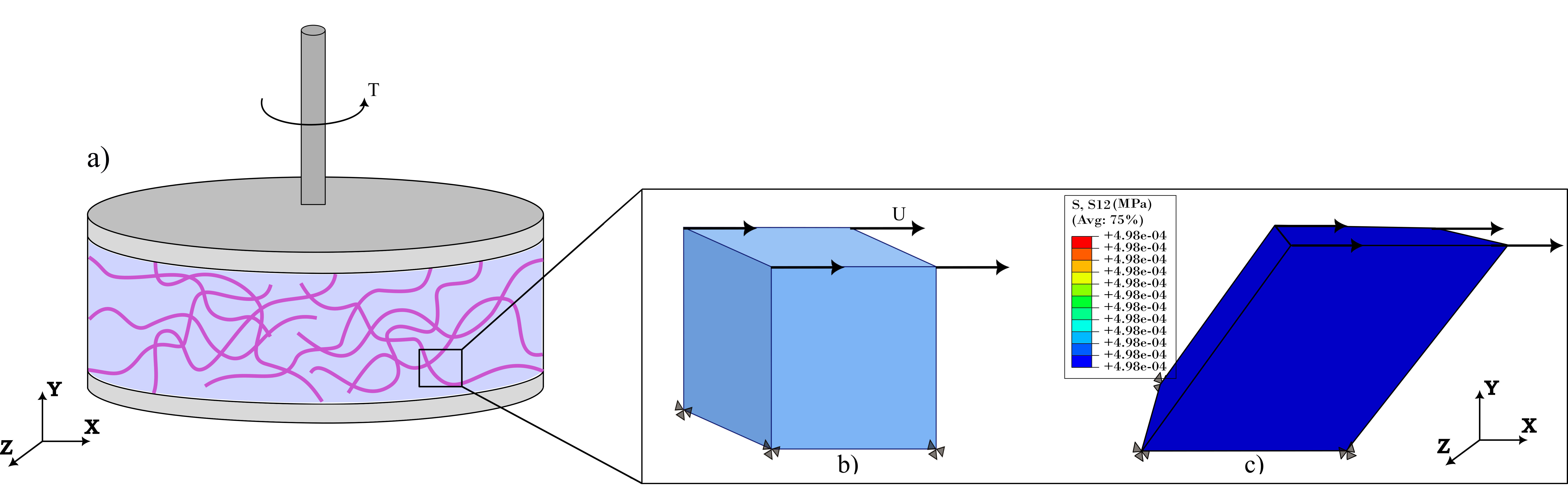

Supplement: Supplementary file 4 — (PNG 883 kb) [file 12195_2026_913_MOESM4_ESM.png]

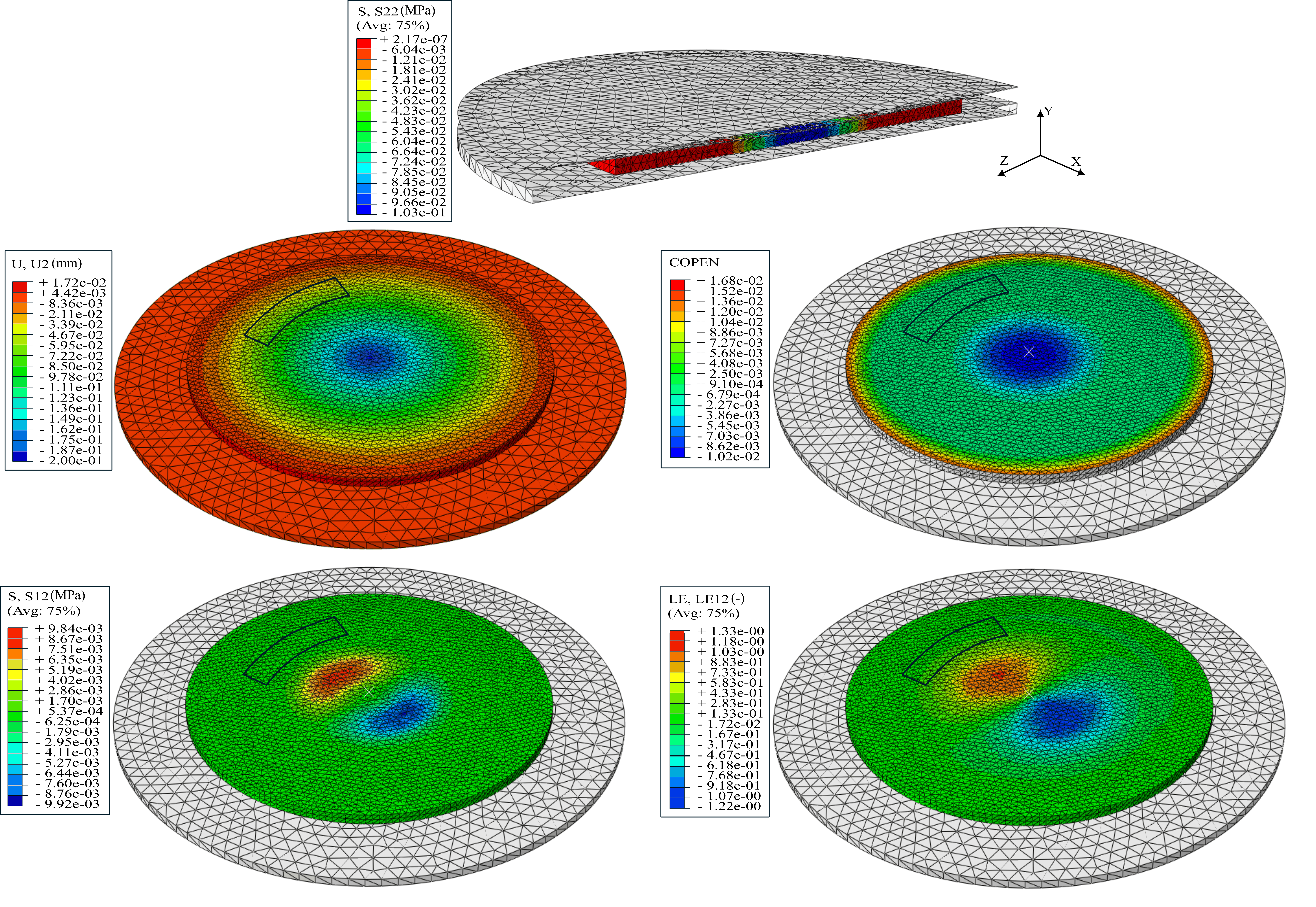

Supplement: Supplementary file 5 — (PNG 21060 kb) [file 12195_2026_913_MOESM5_ESM.png]

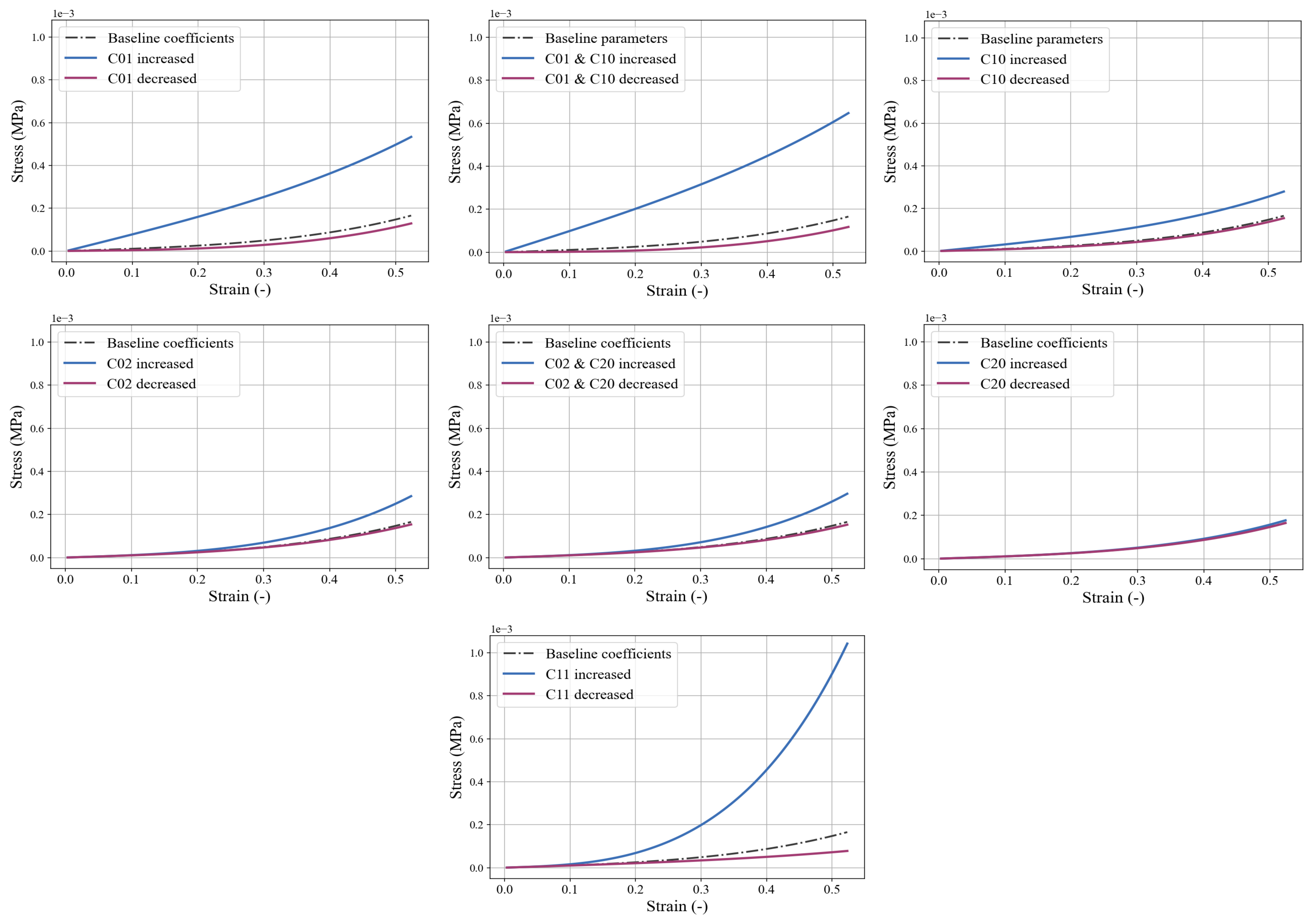

Supplement: Supplementary file 6 — (PNG 1656 kb) [file 12195_2026_913_MOESM6_ESM.png]
